# Cigarette smoking is associated with high HIV viral load among adults presenting for antiretroviral therapy in Vietnam

**DOI:** 10.1371/journal.pone.0173534

**Published:** 2017-03-07

**Authors:** Todd M. Pollack, Hao T. Duong, Thuy T. Pham, Cuong D. Do, Donn Colby

**Affiliations:** 1 The Partnership for Health Advancement in Vietnam (HAIVN), Hanoi, Vietnam; 2 Beth Israel Deaconess Medical Center (BIDMC), Harvard Medical School, Boston, Massachusetts, United States of America; 3 Department of Infectious Diseases, Bach Mai hospital (BMH), Hanoi, Vietnam; 4 Center for Applied Research on Men and Community Health (CARMAH), HCMC, Vietnam; 5 SEARCH, Thai Red Cross AIDS Research Centre, Bangkok, Thailand; CEA, FRANCE

## Abstract

High HIV viral load (VL >100,000 cp/ml) is associated with increased HIV transmission risk, faster progression to AIDS, and reduced response to some antiretroviral regimens. To better understand factors associated with high VL, we examined characteristics of patients presenting for treatment in Hanoi, Vietnam. We examined baseline data from the Viral Load Monitoring in Vietnam Study, a randomized controlled trial of routine VL monitoring in a population starting antiretroviral therapy (ART) at a clinic in Hanoi. Patients with prior treatment failure or ART resistance were excluded. Characteristics examined included demographics, clinical and laboratory data, and substance use. Logistic regression was used to calculate crude and adjusted odds ratios (aOR) and 95% confidence intervals (95% CI). Out of 636 patients, 62.7% were male, 72.9% were ≥30 years old, and 28.3% had a history of drug injection. Median CD4 was 132 cells/mm3, and 34.9% were clinical stage IV. Active cigarette smoking was reported by 36.3% with 14.0% smoking >10 cigarettes per day. Alcohol consumption was reported by 20.1% with 6.1% having ≥5 drinks per event. Overall 53.0% had a VL >100,000 cp/ml. Male gender, low body weight, low CD4 count, prior TB, and cigarette smoking were associated with high VL. Those who smoked 1–10 cigarettes per day were more likely to have high VL (aOR = 1.99, 95% CI = 1.15–3.45), while the smaller number of patients who smoked >10 cigarettes per day had a non-significant trend toward higher VL (aOR = 1.41, 95% CI = 0.75–2.66). Alcohol consumption was not significantly associated with high VL. Tobacco use is increasingly recognized as a contributor to premature morbidity and mortality among HIV-infected patients. In our study, cigarette smoking in the last 30 days was associated with a 1.5 to 2-fold higher odds of having an HIV VL >100,000 cp/ml among patients presenting for ART. These findings provide further evidence of the negative effects of tobacco use among HIV-infected patients.

## Introduction

The HIV viral load (VL) is an important predictor of HIV disease progression. A VL greater than 100,000 copies/ml (cp/ml) is associated with a slower rate of achieving viral suppression [1], faster progression to AIDS [2–4], and reduced response to some antiretroviral drugs [5]. In addition, the plasma VL correlates with the risk of HIV transmission [6–8]. Individuals presenting to care with a VL >10,000 cp/ml are significantly more likely to have transmitted virus to an uninfected partner than those with a VL <10,000 cp/ml [8]. Suppression of VL with antiretroviral therapy (ART) reduces the risk of HIV transmission to sero-discordant partners by 96% [9], and reducing the average VL of HIV-infected patients in a community correlates with reduction of HIV incidence in that community [10].

A number of factors may be associated with the plasma HIV VL in untreated patients. Higher levels of HIV VL have been found in patients who are male [11–13], older age [14], and among those expressing certain HLA subtypes [15,16]. HIV VL is significantly higher during acute HIV infection and during late stage AIDS when the CD4 cell count is <50 cells/mm^3^ [17]. Co-infection with syphilis [18], and other sexually transmitted infections have been shown to increase the HIV VL of an untreated patient [19]. Behavioral factors such as drug and alcohol use and cigarette smoking may also influence a patient’s HIV VL [20–22].

In Vietnam, current HIV treatment guidelines recommend initiation of ART for patients with CD4 <500 cells/mm^3^ but the majority of patients present late to care with CD4 counts <250 cells/mm^3^ [23]. Due to limited resources, VL testing is generally performed only in those suspected of clinical and/or immunological treatment failure [24] and VL measurement at ART initiation is not routine. To better understand factors associated with HIV high VL, we examined demographic, behavioral (alcohol drinking, cigarette smoking), and clinical characteristics of patients presenting for treatment in Hanoi, Vietnam.

## Methods

### Study population

We used baseline data from the Viral Load Monitoring in Vietnam (VMVN) Study, a prospective, randomized controlled trial of routine VL monitoring versus standard monitoring in a patient population starting ART between 4/2011–4/2014. Standard monitoring included CD4 count testing every six months and targeted VL testing to confirm suspected treatment failure based on the presence of clinical and/or immunological criteria [25]. The intervention group received VL testing every six months in addition to the standard monitoring approach.

The study was conducted at the outpatient clinic (OPC) of Bach Mai Hospital, a national level hospital in Hanoi, Vietnam. Participants were patients registered at the OPC who met the following inclusion criteria: (1) age ≥18 years old; (2) confirmed HIV infection; (3) eligible for ART according to criteria defined by Vietnam Ministry of Health HIV guidelines; and (4) ART-naive (never taken any ART for their HIV infection) or off ART for at least 3 months prior to enrollment. Patients with a history of treatment failure on first-line ART or known resistance to first-line ART were excluded. VL testing was performed using the Roche COBAS^®^ AmpliPrep/COBAS^®^ TaqMan^®^ HIV-1 Test, v2.0 (limits of detection: 20–10^7^ cp/ml).

A total of 657 patients signed the consent form, but nine were excluded for not meeting all eligibility requirements. The remaining sample (n = 648) was randomized into two groups: standard monitoring (n = 344) and VL monitoring (n = 304). Five patients who did not have an adequate specimen for VL testing and seven who did not self-report alcohol drinking and cigarette smoking were excluded (0.77%). The remaining 636 patients were included in this analysis.

The study was approved by the Institutional Review Board of Beth Israel Deaconess Medical Center (#2010P000334) in Boston, USA and the Ethical Committee of Bach Mai Hospital in Hanoi, Vietnam. All subjects provided written informed consent prior to study participation. The VMVN study was registered at www.clinicaltrials.gov (ClinicalTrials.gov identification number: NCT01317498). The study was conducted in accordance with the principles of the Declaration of Helsinki.

### Variables, measures, and definitions

Data collected at baseline from study subjects included age, gender, number of years of education, body mass index (BMI), HIV transmission route, history of tuberculosis (TB), current opportunistic infections (OIs), WHO clinical stage, CD4 cell count, hepatitis B virus surface antigen (HBsAg), hepatitis C virus antibody (anti-HCV), cigarette smoking, and alcohol use.

Subjects were asked about daily cigarette smoking in the last 30 days using the following categories: (1) no smoking; (2) one cigarette per day; (3) 2–10 cigarettes per day; (4) 11–20 cigarettes per day; and (5) >20 cigarettes per day. Due to the small number of individuals in some groups, subjects were categorized as follows: (1) no smoking; (2) smoking ≤10 cigarettes per day; and (3) smoking >10 cigarettes per day. This measurement was previously used by the National Cancer Institute [26] and Centers for Disease Control and Prevention [27].

To assess alcohol use, subjects were asked whether they had any alcohol in the last 30 days, and if so, the number of alcoholic beverages per drinking event: (1) no drinking; (2) 1–2 drinks; (3) 3–4 drinks; and (4) ≥5 drinks. A standard drink was defined as a bottle of beer (about 12 ounces) or a glass of wine (about 4–5 ounces). Drinking status was classified as no drinking, drinking without binging, and binge drinking (least 5 drinks per drinking occasion) in the last 30 days [28,29].

Of those included in this paper, 78.6% (n = 424) reported heterosexual contact as their main transmission route, 17.4% drug injection use (IDU), 0.5% having sex with men (MSM), and 3.5% other. Among those reporting heterosexual contact, 13.8% (n = 69) also reported IDU, and 1.4% MSM (n = 7) as their other possible route. As IDU is more likely to be responsible for HIV transmission, we categorized transmission route as follows: IDU with or without other route (28.3%), heterosexual contact only (67.8%), and other (3.9%).

At enrollment, 14.5% of the patients reported having a history of tuberculosis; majority of them (77.2%) were still on TB treatment, 12.0% had completed the treatment, and 10.8% did not report treatment status. Due to this distribution, we decided to define history of TB as having previous TB regardless of the treatment status. Current OI was recorded by the study doctor during the physical examination at enrollment.

HBV and HCV infections were defined as follows: no evidence of current HBV or HCV infection (HBsAg negative, anti-HCV negative); current HBV infection only (HBsAg positive, anti-HCV negative); current or past HCV infection only (HBsAg negative, anti-HCV positive); and current HBV infection and current or past HCV infection (HBsAg positive, anti-HCV positive). BMI is defined as weight in kg/squared height in meter (kg/m2). Weight status was categorized as underweight–BMI <18.5, healthy weight—18.5 ≤ BMI < 25, overweight or obesity—BMI ≥ 25 [30].

About 90.0% of the participants in this study had a viral load value above 10,000 copies/ml, 68.7% above 50,000 copies/ml, and about 53.0% above 100,000 copies/ml. The studied outcome was high VL defined as VL level >100,000 cp/ml [3,5].

### Data analysis

Percent distributions of the studied variables were displayed. Pearson’s Chi-square or Fisher’s exact test was used to compare proportions between the high and low VL groups. Logistic regression was used to calculate crude and adjusted odds ratios (aOR) and 95% confidence intervals (95% CI) for the associations. Bivariate analyses were used to examine the association between HIV VL and each of the variables (age, gender, education, BMI, cigarette smoking, alcohol drinking, transmission route of HIV, history of TB, OIs, WHO clinical stage, CD4, HBV and HCV infections). All variables of interest were included in the multivariate model, and then insignificant variables were removed using likelihood ratio test [31,32]. Covariates previously reported as factors, such as age and alcohol drinking [14,22], were included in the final model regardless of the results of the likelihood ratio test. All analyses were performed using Stata/SE 13.1 (Stata Corporation, College Station, TX).

## Results

The clinical and demographic characteristics of the study subjects are shown in Table 1. The median age was 33.4 years (range, 18.5–74.1 years). The majority of patients (62.7%) were male. Twenty-five patients (3.9%) had any prior exposure to ART; the remaining 96.1% were ART naïve. A history of injection drug use was reported by 180 (28.3%), alcohol use during the past 30 days was reported by 128 (20.1%) and cigarette smoking during the past 30 days was reported by 231 (36.3%). Ninety-two patients (14.5%) had a history of TB infection, 290 (45.6%) had at least one OI, and 232 (36.6%) were anti-HCV positive. Median CD4 cell count was 132 (range, 0–1116 cells/mm^3^) and 222 (34.9%) were clinical stage IV. Median VL (interquartile range–IQR) at presentation was 114,500 cp/ml (35,900–33,5500) with a range from undetected to >10^7^ cp/ml). Percent distribution of categories of baseline VL was as follows: (1) < 40 cp/ml (0.9%); (2) 40–1000 cp/ml (2.2%); (3) 1,001–10,000 cp/ml (7.9%); (4) 10,001–100,000 cp/ml (36.0%); and (5) >100,000 cp/ml (53.0%).

**Table 1:** Clinical and demographic characteristics of the study subjects by the viral load level (n = 636).

	Total	Low VL ≤10^5^ cp/ml	High VL >10^5^ cp/ml	P value
**Gender**				
Male	399 (62.7)	158 (39.6)	241 (60.4)	<0.001
Female	237 (37.3)	141 (59.5)	96 (40.5)	
**Age (yrs)**				
<25	51 (8.0)	29 (56.9)	22 (43.1)	0.121
25-<30	121 (19.0)	56 (46.3)	65 (53.7)	
30-<35	212 (33.3)	108 (50.9)	104 (49.1)	
35+	252 (39.6)	106 (42.1)	146 (57.9)	
**Education (yrs)**				
≤5	42 (6.6)	16 (38.1)	26 (61.9)	0.581
6–9	209 (32.9)	97 (46.4)	112 (53.6)	
10–12	270 (42.5)	133 (49.3)	137 (50.7)	
>12	115 (18.1)	53 (46.1)	62 (53.9)	
**Body mass index (BMI)**				
Underweight (<18.5)	214 (33.6)	69 (32.2)	145 (67.8)	<0.001
Normal or healthy weight (18.5–24.9)	394 (61.9)	209 (53.0)	185 (47.0)	
Overweight or obese (≥25.0)	28 (4.4)	21 (75.0)	7 (25.0)	
**Cigarette smoking in the last 30 days**				
No smoking	405 (63.7)	208 (51.4)	197 (48.6)	0.004
Smoking ≤10 cigarettes per day	142 (22.3)	50 (35.2)	92 (64.8)	
Smoking >10 cigarettes per day	89 (14.0)	41 (46.1)	48 (53.9)	
**Alcohol drinking in the last 30 days**				
No drinking	508 (79.9)	232 (45.7)	276 (54.3)	0.244
Drinking, not binge (<5 drinks per occasion)	89 (14.0)	44 (49.4)	45 (50.6)	
Binge drinking (≥5 drinks per occasion)	39 (6.1)	23 (59.0)	16 (41.0)	
**HIV transmission route**				
Heterosexual contact	431 (67.8)	213 (49.4)	218 (50.6)	0.172
Drug injection	180 (28.3)	77 (42.8)	103 (57.2)	
Other	25 (3.9)	9 (36.0)	16 (64.0)	
**History of tuberculosis (TB)**				
No	544 (85.5)	276 (50.7)	268 (49.3)	<0.001
Yes	92 (14.5)	23 (25.0)	69 (75.0)	
**Current opportunistic infections (OIs)**				
No	346 (54.4)	216 (62.4)	130 (37.6)	<0.001
Yes	290 (45.6)	83 (28.6)	207 (71.4)	
**WHO clinical stage**				
I	297 (46.7)	192 (64.6)	105 (35.4)	<0.001
II	48 (7.5)	20 (41.7)	28 (58.3)	
III	69 (10.8)	27 (39.1)	42 (60.9)	
IV	222 (34.9)	60 (27.0)	162 (73.0)	
**CD4 (cells/mm**^**3**^**)**				
≤100	293 (46.1)	69 (23.5)	224 (76.5)	<0.001
101–250	141 (22.2)	74 (52.5)	67 (47.5)	
>250	202 (31.8)	156 (77.2)	46 (22.8)	
**Hepatitis**				
HBs Ag (-) and anti-HCV (-)	361 (56.9)	179 (49.6)	182 (50.4)	0.182
HBs Ag (+) only	41 (6.5)	21 (51.2)	20 (48.8)	
Anti-HCV (+) only	192 (30.3)	78 (40.6)	114 (59.4)	
HBs Ag (+) and anti-HCV (+)	40 (6.3)	21 (52.5)	19 (47.5)	

On bivariate analysis (Table 1) multiple factors were associated with a high baseline HIV VL, including male gender (p <0.001), low body weight (p <0.001), history of TB (p <0.001), low CD4 cell count (p <0.001), current OI (p <0.001), advanced clinical stage (p <0.001), and cigarette smoking (p = 0.004). Self-reported alcohol use, self-reported injection drug use, lower education level, older age, HBsAg, and anti-HCV were not significantly associated with baseline VL.

The results of the multivariate analysis are shown in Table 2. After controlling for other variables, male patients were more likely to have a high VL compared to female patients (aOR = 1.62, 95% CI: 0.96–2.71). In addition, cigarette smoking was also independently associated with high baseline VL. Those who smoked 1–10 cigarettes per day were twice as likely to have a high VL as those who had never smoked (aOR = 1.99, 95% CI: 1.15–3.45). A smaller number of patients who smoked more than 10 cigarettes per day were also more likely to have a high VL but the association did not reach statistical significance (aOR = 1.41, 95% CI: 0.75–2.66). Having prior TB was also associated with a high HIV VL (aOR = 1.88, 95% CI: 1.05–3.36).

**Table 2:** Factors associated with high HIV viral load (>10^5^ cp/ml) at enrollment.

	Crude ORs (95% CI)	Adjusted ORs (95% CI)
**Gender**		
Male	2.24 (1.61–3.11)	1.62 (0.96–2.71)
Female	1	1
**Age (yrs)**		
<25	0.55 (0.30–1.01)	1.00 (0.49–2.06)
25-<30	0.84 (0.54–1.30)	1.25 (0.73–2.14)
30-<35	0.70 (0.48–1.01)	0.83 (0.54–1.29)
35+	1	1
**Body mass index (BMI)**		
Underweight (<18.5)	2.37 (1.68–3.36)	1.43 (0.94–2.18)
Normal or healthy weight (18.5–24.9)	1	1
Overweight or obese (≥25.0)	0.38 (0.16–0.91)	0.41 (0.16–1.10)
**Cigarette smoking in the last 30 days**		
No smoking	1	1
Smoking ≤10 cigarettes per day	1.94 (1.31–2.89)	1.99 (1.15–3.45)
Smoking >10 cigarettes per day	1.24 (0.78–1.96)	1.41 (0.75–2.66)
**Alcohol drinking in the last 30 days**		
No drinking	1	1
Drinking, not binge (<5 drinks per occasion)	0.86 (0.55–1.35)	0.90 (0.51–1.59)
Binge drinking (≥5 drinks per occasion)	0.58 (0.30–1.13)	0.66 (0.29–1.50)
**History of tuberculosis (TB)**		
No	1	1
Yes	3.09 (1.87–5.10)	1.88 (1.05–3.36)
**CD4 (cells/mm**^**3**^**)**		
≤100	1	1
101–250	0.28 (0.18–0.43)	0.31 (0.20–0.50)
>250	0.09 (0.06–0.14)	0.11 (0.07–0.17)
**Hepatitis**		
HBs Ag (-) and anti-HCV (-)	1	1
HBs Ag (+) only	0.94 (0.49–1.79)	0.54 (0.25–1.18)
Anti-HCV (+) and HBs Ag (+)/(-)	1.32 (0.95–1.85)	0.49 (0.30–0.79)

CD4 cell count and body weight were inversely related to VL. Compared to those with a CD4 <100 cells/mm^3^, patients with a CD4 >250 cells/mm^3^ were one-tenth as likely to have a VL greater than 100,000 cp/ml (aOR = 0.11, 95% CI: 0.07–0.17). In contrast to the finding in the bivariate analysis, multivariate analysis showed that patients with a positive HCV antibody were less likely to have a high HIV VL (aOR = 0.49, 95% CI: 0.30–0.79). Alcohol consumption was not significantly associated with a high VL after controlling for other factors (aOR = 0.90, 95% CI: 0.51–1.59 for those with <5 drinks per event, and aOR = 0.66, 95% CI: 0.29–1.50 for those with 5 or more drinks per event).

## Discussion

In this cohort of patients presenting for ART in Hanoi, Vietnam, we found that more than half of the patients presented with a VL >100,000 cp/ml. This finding is consistent with a high prevalence of late presentation to care in Vietnam [33]. Despite expanding access to ART in the country, the median CD4 cell count at ART initiation was 197 cells/mm^3^ in 2013 [23,34]. In our study, 68% presented with CD4 <250 cells/mm^3^ and 35% with clinical stage IV. Baseline VL testing is not recommended for patients in Vietnam according to the Vietnam’s National HIV Guidelines [24].

We found an association between cigarette smoking and VL at time of ART initiation. Daily smokers were twice as likely to have a VL >100,000 cp/ml than nonsmokers. This association remained after controlling for other factors such as age, gender, body weight, alcohol use, history of TB, CD4 cell count, and HBV and HCV infection. Those who smoked 1–10 cigarettes per day were more likely to have high VL (aOR = 1.99, 95% CI = 1.15–3.45). The association among those who smoked >10 cigarettes per day was of borderline statistically significance (aOR = 1.41, 95% CI = 0.75–2.66), which may be explained by the smaller number of patients in this category. This finding is consistent with a recent study of adults in Cameroon which demonstrated a 1.4 log increase in the plasma VL of HIV positive smokers compared to nonsmokers [20]. Tobacco use has also been shown to affect the response to ART. Smoking was associated with failure to achieve a suppressed HIV VL in a study of HIV patients in the US [21]. Moreover, Miguez-Burbano et al. reported that the immune and virological response to ART was attenuated by 40% among daily tobacco users [35]. Other studies also reported a positive association between current smoking and detectable VL [36], and a higher risk of virologic rebound among smokers compared to non-smokers [37]. In contrast, a study of HIV infected persons with alcohol problems in the US did not support a positive association between smoking cigarettes and HIV VL [38].

Ande et al. showed a significant increase in oxidative stress among HIV-infected individuals and smokers compared with HIV negative non-smokers, suggesting that oxidative stress and induction of the cytochrome P450 (CYP) pathway could explain the mechanism of smoking-related increased VL among HIV infected individuals [20]. Similarly, Feldman et al. found that homozygosity for the CYP1A1-m1 polymorphism was associated with impaired viral response to ART among smokers, but that it had no effect among nonsmokers. The authors speculated that the enzyme coded by CYP1A1-m1 may increase HIV VL through conversion of toxins found in cigarette smoke into DNA adducts, which may directly promote HIV-1 gene expression [39].

Our study did not demonstrate an association between alcohol consumption and HIV VL at ART initiation. Similar findings have been reported by other investigators [22,40–43]. In the study of Samet et al., heavy alcohol consumption was not associated with HIV RNA in both ART and pre-ART patients [43]. Baum et al. reported an association between frequent alcohol intake and VL among those receiving ART, but not in those without ART [22]. These authors hypothesize that alcohol abuse reduces adherence to ART, resulting in an increase in VL. Although heavy drinkers were found to be more likely to interrupt ART, the same study reported no effect of alcohol consumption on VL in both groups of ART-initiating and ART-naive individuals [42].

Consistent with findings from previous reports [11–13], our study found that males were more likely to have a high VL at ART initiation than female patients. Women tend to present earlier for care compared to men and a later presentation for ART initiation could contribute to differences in VL at presentation. However, in our study, the higher VL among men remained after controlling for differences in baseline CD4 counts. Inherent gender biological differences may partially explain gender differences in VL. Female hormones such as progesterone inhibit C-chemokine receptor 5 (CCR5) expression on activated T cells CD4, which in turn lowers the VL [12].

In contrast, we did not find an association between age and baseline HIV VL after controlling for other factors. This is inconsistent with other studies showing that older HIV-infected adults tend to present at a more advanced stage of infection (49) and have a more rapid rise of HIV viral load over time (50). The lack of association in our study may be explained by the relatively few patients who were greater than 50 years old in our cohort. While certainly aging, the HIV patient population in Vietnam is still relatively young compared to countries with more mature epidemics. In our cohort, only 7% of patients were 50 years old or greater.

High HIV VL was more likely to be found in patients with other markers of advanced HIV disease such as low CD4, low body weight, and current opportunistic infections. Patients with clinical stage III or IV disease at presentation were more likely to have a VL > 100,000 then those with clinical stage I or II. This is consistent with well-established evidence that plasma HIV VL is associated with more rapid declines in CD4 cells and an increased risk of progression to symptomatic disease and AIDS [3,44]. Opportunistic infections and TB infection, in particular, has been shown to be associated with increases in plasma HIV viremia [45–47]. Goletti et al. observed a 5- to 160-fold increase in plasma HIV VL in patients with active TB with a subsequent reduction of VL following successful TB treatment [48]. Immune activation, induced from antigen-specific response against mycobacterium tuberculosis, may drive the increase in HIV viral replication. In our study, patients with TB co-infection were nearly twice as likely to have high VL.

Patients in our cohort with a positive antibody against HCV were less likely to have a high VL (OR = 0.49, 95% CI = 0.30–0.79). An inverse association between HIV VL and HCV status was also found in a cross-sectional study of HIV-infected Vietnamese patients who did not meet criteria for ART [13]. Studies of HBV and HCV co-infection have suggested the possibility of a reciprocal interaction between HCV infection and HBV infection [49–51]. Zarski et al. found significantly lower HCV RNA levels among patients with detectable HBV DNA compared to HBV DNA negative patients, suggesting that active HBV co-infection is associated with decreased replication of HCV RNA [49]. Chakravarti et al. showed an inverse association between HCV infection and the replication of HBV or HBV DNA [50] and French et al. showed ongoing HCV replication, but not resolved HCV infection, was associated with isolated anti-HBc without viremia (HBsAg-, anti-HBs-) [51]. The authors speculated that the association might be due to a biological interaction between HCV infection and HBV infection or between HCV infection and the immunologic response to HBV infection [51]. However, a similar interaction between HIV and HCV has not been described.

The VMVN study is one of the few studies in Vietnam performing HIV VL testing for a large number of patients who were eligible for ART based on clinical and immunological criteria. However, several limitations exist. First, patients were asked to estimate smoking and alcohol consumption in the past month so recall and social desirability biases are possible. We cannot exclude the potential effect of smoking history beyond the last 30 days. Patients presenting with advanced HIV disease might reduce their smoking and alcohol intake due to worsening health status. However, in that case, the association between smoking and high HIV viral load would be underestimated. Our data showed a slightly lower alcohol use among patients with stage IV disease, but smoking was not significantly associated with disease stage. Second, although a single question on the quantity of smoking and alcohol use may not provide the best estimate, it is useful for comparative purposes [52]. Third, we cannot exclude the potential effect of unmeasured confounders on our findings. However, our analysis did include the most common factors associated with viral load in other studies. Fourth, the study recruited patients from a hospital-based clinic where patients may present in a more advanced stage of HIV infection than those who enrolled in community-based clinics.

## Conclusions

In this cohort of patients with criteria for ART initiation, we found that male gender, advanced HIV disease (low CD4, low body weight, prior TB), and daily cigarette smoking in the last 30 days increased the odds of having a high VL. Daily cigarette smoking in the last 30 days was associated with a 1.5 to 2-fold higher odds of having a VL >100,000 cp/ml. Tobacco use is increasingly recognized as a significant contributor to premature morbidity and mortality among HIV-infected patients. These findings provide further evidence of the negative effects of tobacco use among HIV-infected patients.

## Supporting information

S1 Dataset (DTA)
